# ARRB1 Regulates Metabolic Reprogramming to Promote Glycolysis in Stem Cell-Like Bladder Cancer Cells

**DOI:** 10.3390/cancers13081809

**Published:** 2021-04-10

**Authors:** Kenza Mamouni, Jeongheun Kim, Bal L. Lokeshwar, Georgios Kallifatidis

**Affiliations:** 1 Georgia Cancer Center, Augusta University, Augusta, GA 30912, USA; kmamouni@augusta.edu (K.M.); jeokim@augusta.edu (J.K.); 2 Research Service, Charlie Norwood VA Medical Center, Augusta, GA 30904, USA; 3 Department of Biological Sciences, Augusta University, Augusta, GA 30912, USA

**Keywords:** bladder cancer, cancer system cells, metabolic reprograming, β-arrestin 1, mitochondrial pyruvate carrier, glucose transporter

## Abstract

**Simple Summary:**

Bladder cancer (BC) ranks second in incidence and mortality among all genitourinary cancers. The high recurrence of BC is attributed to the presence of cancer stem cells (CSCs), which are the driving force behind tumor growth. Increasing evidence suggests that stem cells exhibit a distinct metabolic program compared to differentiated cells. Understanding their metabolic preference for maintaining stem cell properties is essential for developing novel therapeutics targeting CSCs. The current work shows for the first time that the scaffold protein β-arrestin1 (ARRB1) functions as a metabolic switch regulating the metabolic reprogramming of CSC-like cells towards glycolysis by regulating the mitochondrial pyruvate carrier MPC1 and glucose transporter GLUT1. The balance between glycolysis and oxidative phosphorylation plays a crucial role in regulating the fate of stem cells. Our findings will potentially open new therapeutic avenues for targeting bladder cancer cells and/or the CSC-like cells within aggressive bladder tumors.

**Abstract:**

β-arrestin 1 (ARRB1) is a scaffold protein that regulates signaling downstream of G protein-coupled receptors (GPCRs). In the current work, we investigated the role of ARRB1 in regulating the metabolic preference of cancer stem cell (CSC)-like cells in bladder cancer (BC). We show that ARRB1 is crucial for spheroid formation and tumorigenic potential. Furthermore, we measured mitochondrial respiration, glucose uptake, glycolytic rate, mitochondrial/glycolytic ATP production and fuel oxidation in previously established ARRB1 knock out (KO) cells and corresponding controls. Our results demonstrate that depletion of ARRB1 decreased glycolytic rate and induced metabolic reprogramming towards oxidative phosphorylation. Mechanistically, the depletion of ARRB1 dramatically increased the mitochondrial pyruvate carrier MPC1 protein levels and reduced the glucose transporter GLUT1 protein levels along with glucose uptake. Overexpression of ARRB1 in ARRB1 KO cells reversed the phenotype and resulted in the upregulation of glycolysis. In conclusion, we show that ARRB1 regulates the metabolic preference of BC CSC-like cells and functions as a molecular switch that promotes reprogramming towards glycolysis by negatively regulating MPC1 and positively regulating GLUT1/ glucose uptake. These observations open new therapeutic avenues for targeting the metabolic preferences of cancer stem cell (CSC)-like BC cells.

## 1. Introduction

Bladder cancer (BC) ranks second in incidence and mortality among all genitourinary cancers, and mortality remains unchanged over the last 25 years. Frequent recurrence is not only associated with morbidity and mortality in BC, but also has staggering economic implications. The annual cost of treating BC patients in the US is greater than $100 million [1,2]. The high recurrence of BC is attributed to the presence of cancer stem cells (CSCs) in bladder tumors. CSCs are implicated in therapeutic resistance and tumor relapse after initial therapy in various cancer types. Understanding how CSCs differ from bulk cancer cells and how they contribute to relapse and resistance is essential to develop novel therapeutics to target CSCs effectively.

Increasing evidence suggests that stem cells exhibit a distinct metabolic program [3,4,5] compared to differentiated cells. Consistently, the metabolic preference of CSCs differs from those of tumor bulk cells. The balance between glycolysis and oxidative phosphorylation plays a crucial role in regulating the fate of stem cells. Some stem cells, including hematopoietic stem cells (HSCs) and induced pluripotent cells (iPSCs), rely on glycolysis for energy generation [6,7,8,9,10]. However, muscle stem cells, as well as pancreatic CSCs, rely on oxidative phosphorylation for ATP generation [11,12,13]. Thus, there is no unifying model for the metabolic preference of stem cells and only very little research has focused on how metabolic reprogramming regulates the stem cell pool and maintenance of stemness. Understanding the metabolic program required for maintaining stem cell properties (such as self-renewal and multilineage differentiation potential) will open new therapeutic avenues for specifically targeting CSCs, which are implicated in tumor initiation, relapse, metastases, and therapy resistance.

The metabolic fate of the energetic substrate pyruvate plays a key role in homeostasis between glycolysis and oxidative phosphorylation. The transporters that facilitate the import of pyruvate in the mitochondria were first described in 2012 as mitochondrial pyruvate carrier 1 (MPC1) and mitochondrial pyruvate carrier 2 (MPC2) [14,15]. The MPCs, which dictate the metabolic fate of cells, have been recently shown to regulate several CSC markers as well as self-renewal. The depletion and inhibition of MPC1 were shown to induce a metabolic reprogramming towards aerobic glycolysis that was accompanied by an upregulation of stem cell markers ALDH and CD44, and an increase in migratory potential and resistance towards chemotherapy in prostate cancer [16]. Consistently, the overexpression of MPC1 reduced stem cell marker expression and inhibited spheroid growth, indicating the inhibition of self-renewal [16,17].

β-Arrestins are well-known negative regulators of G-protein-coupled receptor (GPCR) signaling. β-arrestin binding to GPCRs both uncouples receptors from heterotrimeric G proteins and targets them to clathrin coated pits for endocytosis. However, in certain circumstances, β-arrestins can function as adaptor molecules that mediate G-protein independent signaling by serving as scaffolds that link signaling networks [18]. Our previous work shows that β-arrestins regulate stem cell properties in BC [19]. We have demonstrated that ARRB1 regulates the CSC markers CD44 and Bmi1 [19]. The latter is a transcription factor involved in self-renewal and regulation of ROS [20].

In the present work, we analyzed for the first time the role of adapter protein ARRB1 in regulating the metabolic preference of CSC-like bladder cancer cells. We knocked out ARRB1 to investigate whether ARRB1 can promote metabolic reprogramming towards glycolysis. We further investigated how ARRB1 dictates the metabolic preference of BC cells by looking at its effects on key proteins (e.g., MPC1) at the crossroad between glycolysis and oxidative phosphorylation. Our findings will potentially open new therapeutic avenues for targeting bladder cancer cells and/or the CSC-like cells within aggressive bladder tumors.

## 2. Materials and Methods

### 2.1. Cell Culture and Transfection

Bladder cancer cell lines (HT1376, T24 and 5637) were obtained from American Type Culture Collection (Rockville, MD, USA) and were used within 10 passages. The 253J cells and nonmalignant bladder epithelial cells (UROTSA) were a kind gift of Dr. Colin Dinney (MD Anderson Cancer Center, Houston, TX, USA) and Dr. Donald Sens (University of North Dakota, Grand Forks, ND, USA), respectively. All of the above-described cells were cultured in RPMI medium supplemented with 10% FBS and gentamicin and cultured in a humidified incubator with 5% CO_2_ at 37 °C. Fetal bovine serum (FBS) was purchased from Atlanta Biologicals (Atlanta, GA, USA), antibiotics (gentamycin) and cell culture media were purchased from Invitrogen (Carlsbad, CA, USA) or Sigma-Aldrich (St. Louis, MO, USA). For over expression of ARRB1 cells were transfected with an ARRB1 plasmid (SC303424, Origene, Rockville, MD, USA) using Lipofectamine 3000 (L3000001, Thermo Fisher Scientific, Waltham, MA, USA).

### 2.2. Spheroid Formation Assays

Clonogenic growth was measured using colony formation assays as previously described [21]. Tumor spheroid formation was determined by culturing cells at low density (2000 cells/well) on SeedEZ^TM^ 3D scaffolds (Z742246-24EA, Lena Biosciences, Atlanta, GA, USA) placed in white 96-well plates in SpheroMax medium and supplement (C-28070, PromoCell; Heidelberg, Germany). Spheroid viability was analyzed 72 h later utilizing Celltiter-Glo 3D cell viability assay (G968A, Promega, Madison, WI, USA) according to the manufacturer’s instructions.

### 2.3. Immunoblotting

Cell lysates were prepared using the RIPA-buffer supplemented with protease inhibitors (Thermo Scientific 1861281), and total protein was quantified with the Pierce Micro BCA Protein Assay Kit (Thermo Scientific; 23235). Western blot analysis was performed as previously described [22,23]. Expression of β-actin served as a loading control [24]. Antibodies and dilutions are provided in Table S1: Antibodies and dilutions.

### 2.4. Immunofluorescence

We evaluated the expression of CD44 and Bmi-1 in HT1376 derived subcutaneous tumors by immunofluorescence staining. Paraffin-embedded tissue sections were boiled in 10 mM sodium citrate solution +0.05% Tween-20 for 30 min (antigen retrieval), blocked in 3% BSA, 10% goat serum (1 h) and incubated with anti-CD44, or anti-Bmi-1 primary antibodies. Following washing with PBS, the sections were incubated with a fluorophore (Alexa Fluor 488, or Alexa Fluor 546/555) labeled secondary antibodies (Thermo Fisher). Nuclei were stained with DAPI. Labeled cells were observed under a confocal microscope (BZ-X710, Keyence Corporation of America, Itasca, IL, USA).

### 2.5. Glycolysis Cell-Based Assay

A colorimetric assay was used to quantify secreted L-lactate as a measure for glycolysis (600450, Cayman CHEMICAL, Ann Arbor, MI, USA). The assay is based on the chemical reaction between lactate and NAD+ that is catalyzed by lactate dehydrogenase. Produced NADH reduces a tetrazolium salt to a colored product that absorbs light at 490 nm. ARRB1 wildtype (transfected with No-Target plasmid, NT) and ARRB1 knock out (KO) HT1376 cells (KO clone 9 and clone 10) were seeded in 96-well plates in RPMI media with 0.75% FBS and incubated in a CO_2_ incubator for 24 h according to the recommendations of the manufacturer (600450, Cayman CHEMICAL). We centrifuged 96-well plates at 1000 rpm, 5 min. We transferred 10 µL supernatant from each well to a new plate and diluted in 90 µL assay buffer. We added 100 µL reaction solution to each well followed by an incubation for 30 min at RT. Absorbance was read at 490 nm. Measurements were normalized to total cellular protein quantified by Micro BCA Protein Assay Kit (Fisher Scientific, Cat #23235) or cell number.

### 2.6. Measurement of Mitochondrial/Cytoplasmic Pyruvate Ratio

ARRB1 wildtype and ARRB1 KO HT1376 cells were seeded and expanded in cell culture flasks. Cells were collected at a confluence of 80% and mitochondrial and cytosolic fractions from 5 × 10^7^ cells were isolated according to the instructions of the manufacturer (Mitochondrial/Cytosol Fractionation Kit, Biovision, Cat # K256-25, Milpitas, CA, USA). Briefly, cells were centrifuged at 600× *g* for 5 min and washed with ice-cold PBS. The cell pellet was resuspended in 1 × Cytosol Extraction Buffer Mix in presence of protease inhibitors (in absence of DTT), incubated for 10 min on ice and homogenized utilizing an ice-cold dounce tissue grinder. The homogenate was first centrifuged at 700× *g* for 10 min, and the supernatant was further centrifuged at 10,000× *g* for 30 min. The supernatant contained the cytoplasmic protein fraction and the pellet was resuspended in Mitochondrial Extraction Buffer Mix in presence of protease inhibitors (in absence of DTT) and represented the mitochondrial fraction.

Pyruvate levels of mitochondrial and cytosolic fractions were quantified using a Pyruvate Colorimetric Assay Kit (K609-100, Biovision, Milpitas, CA, USA) according to the instructions of the manufacturer. The assay is based on the oxidation of pyruvate by pyruvate oxidase generating a colored product that absorbs light at 570 nm. We diluted 5 µL of mitochondrial or cytosolic extracts in 45 µL of Pyruvate Assay Buffer per well of a 96-well plate followed by the addition of 50 µL/well Reaction mix. Absorbance (570 nm) was measured after an incubation of 30 min and is directly proportional to the pyruvate concentration of the samples. In parallel experiments we measured the ratio of mitochondrial vs. total protein. Protein levels were quantified by Micro BCA Protein Assay Kit (23235, Fisher Scientific).

### 2.7. Glucose Uptake Assay

The Glucose Uptake-Glo™ Assay (J1341, Promega) was utilized to measure glucose uptake in ARRB1 KO cells and corresponding controls. We seeded 20,000 cells/well in a 96-well plate and grown overnight. Media were removed and cells were washed with PBS followed by addition of 50 µL/well of 1 mM 2-deoxyglucose (2DG) solution and incubation for 10 min at room temperature. Once transported into cells, 2DG becomes rapidly phosphorylated to 2DG6P, which was quantified based on the instructions of the manufacturer.

### 2.8. Seahorse: Oxygen Consumption/Extracellular Acidification Rate

#### 2.8.1. Mito Stress Assay

Agilent Seahorse XF Cell Mito Stress Test Kit (Agilent Technologies, Cedar Creek, TX, USA, Cat # 103015) was utilized to determine oxygen consumption rates of HT1376 NT (transfected with No-Target control plasmids, wild type ARRB1) and HT1376 ARRB1 KO cells (clone 9 and clone 10). In an initial experiment, we seeded cells at different densities to determine optimal cell numbers for the Mito stress assay. For all other experiments we plated 10,000 cells/well (in 180 µL) in the Agilent Seahorse Cell Culture Plate. The cells were incubated for 1 h at RT and then overnight in the CO_2_ incubator at 37 °C. The following day, cells were washed and supplied with assay medium (XF Base medium, 10 mM glucose, 1 mM pyruvate, 2 mM glutamine, pH 7.4) and run with the XF96e Analyzer. The Seahorse cartridge was humidified with sterile water overnight in non-CO_2_ incubator at 37 °C and the submerged in prewarmed XF-calibrant solution for 45–60 min. Oligomycin, FCCP and Rotenone/antimycin A solutions were prepared according to the recommendations of the manufacturer and added to cartridge port A, B, or C for a final concentration of 1 µM, 2 µM and 0.5 µM, respectively. Oligomycin targets mitochondrial respiration by inhibiting the ATP synthase. FCCP is a protonophore that collapses the proton gradients in the inner mitochondrial membrane and functions as a mitochondrial oxidative phosphorylation uncoupler. Rotenone and Antimycin A shut down mitochondrial respiration by inhibiting complex I and complex III, respectively. Oxygen consumption measurements were normalized to total cellular protein quantified by Micro BCA Protein Assay Kit (23235, Fisher Scientific) or cell number. Results were analyzed in the WAVE software and processed by the Seahorse XF Cell Mito Stress Test Generator. The measurements were also used to calculate ATP production according to the instructions of the manufacturer. The decrease in oxygen consumption rate following inhibition of the ATP synthase with oligomycin reflects the basal mitochondrial ATP production.

#### 2.8.2. Glycolytic Rate Assay

Agilent Seahorse XF Glycolytic Rate Assay Kit (Agilent Technologies, Cedar Creek, TX, USA, Cat # 103344) was utilized to determine glycolytic rates of HT1376 NT (wild type ARRB1) and HT1376 ARRB1 KO cells (clone 9 and clone 10). We plated 10,000 cells (in 180 µL) per well in the Agilent Seahorse Cell Culture Plate. Cells were incubated for 1 h at RT and then overnight in the CO_2_ incubator at 37 °C. The following day, the cells were washed and supplied with assay medium (XF RPMI medium, 10 mM glucose, 1 mM pyruvate, 2 mM glutamine, pH 7.4) and analyzed with the XF96e Analyzer (Agilent). The Seahorse cartridge was humidified with sterile water overnight in a non-CO_2_ incubator at 37 °C and then submerged in prewarmed XF-calibrant solution for 45–60 min. Rotenone/Antimycin A and 2-deoxy-glucose (2-DG) solutions were prepared according to the recommendations of the manufacturer and added to cartridge port A or B for a final concentration of 0.5 µM and 50 mM, respectively. 2-DG is glucose analog that competitively binds to the glycolytic enzyme hexokinase inhibiting glycolysis. Oxygen consumption rate (OCR) and extracellular acidification rate (ECAR) were measured, and the glycolytic proton efflux rate (PER) was calculated in the WAVE software and processed with the Seahorse XF Glycolytic Rate Assay Generator according to the instructions of the manufacturer. The calculated rates account for contribution of citric acid cycle-derived CO_2_ to extracellular acidification and reflect glycolysis. Measurements were normalized to total cellular protein quantified by Micro BCA Protein Assay Kit (Fisher Scientific, Cat #23235) or cell number.

#### 2.8.3. ATP Rate Assay

Agilent Seahorse XF Real-Time ATP Rate Assay Kit (103592, Agilent Technologies, Cedar Creek, TX, USA) was utilized to discriminate between glycolytic and mitochondrial ATP production in HT1376 NT (wild type ARRB1) and HT1376 ARRB1 KO cells (clone 9 and clone 10). We plated 20,000 cells (in 180 µL) per well in the Agilent Seahorse Cell Culture Plate. The cells were incubated for 1 h at RT and then overnight in the CO_2_ incubator at 37 °C. The following day cells were washed and supplied with assay medium (XF RPMI medium, 10 mM glucose, 1 mM pyruvate, 2 mM glutamine, pH 7.4) and analyzed with the XF96e Analyzer (Agilent). The Seahorse cartridge was humidified with sterile water overnight in a non-CO_2_ incubator at 37 °C and then submerged in prewarmed XF-calibrant solution for 45–60 min. Oligomycin and Rotenone/Antimycin A solutions were prepared according to the recommendations of the manufacturer and added to cartridge port A or B for a final concentration of 1.5 µM and 0.5 µM (0.5 µM, for each Rotenone and antimycin A), respectively. OCR and ECAR were measured and the mitochondrial and glycolytic ATP production were calculated in WAVE software with the Seahorse XF Real-Time ATP Rate Assay Report Generator (Agilent Technologies, Cedar Creek, TX, USA). Measurements were normalized to total cellular protein quantified by Micro BCA Protein Assay Kit (23235, Fisher Scientific) or cell number.

#### 2.8.4. Mitochondrial Fuel Usage Assay

The assay was performed according to the instructions of the manufacturer (103260-100, Agilent Seahorse XF Mito Fuel Flex Test Kit, Agilent Technologies, Cedar Creek, TX, USA). We plated 10,000 cells (in 180 µL) per well in the Agilent Seahorse Cell Culture Plate. Cells were incubated for 1 h at RT and then overnight in the CO_2_ incubator at 37 °C. The following day cells were washed and supplied with assay medium (XF RPMI medium, 10 mM glucose, 1 mM pyruvate, 2 mM glutamine, pH 7.4) and analyzed with the XF96e Analyzer (Agilent). The Seahorse cartridge was humidified with sterile water overnight in a non-CO_2_ incubator at 37 °C and then submerged in prewarmed XF-calibrant solution for 45–60 min. BPTES (inhibitor of the glutaminase/glutamine oxidation pathway), Etomoxir Sodium Salt hydrate (inhibitor of carnitine palmitoyl-transferase 1 a/ long-chain fatty acid oxidation), and UK-5099 (inhibitor of mitochondrial pyruvate carrier (MPC)/ glucose oxidation pathway) were purchased from Sigma (Cat # E1905, SML0601, and PZ0160). Stock solutions were prepared and added to cartridge port A or B according to the recommendations of the manufacturer (Agilent Seahorse XF Mito Fuel Flex Test Kit Manual, Cat # 103260-100, Agilent Technologies, Cedar Creek, TX, USA). OCR was measured, and fuel dependency of cells to oxidize Glucose (pyruvate), Glutamine (Glutamate), and long-chain fatty acids were analyzed in WAVE software and processed through the Seahorse XF Mito Fuel Flex Report generator (Agilent Technologies, Cedar Creek, TX, USA). Measurements were normalized to total cellular protein quantified by Micro BCA Protein Assay Kit (Fisher Scientific, Cat #23235) or cell number.

### 2.9. CRISPR/Cas9

ARRB1 knock out cells were generated by CRISPR/Cas9 genome editing technology and evaluated as described in our previous work [19].

### 2.10. Subcutaneous Xenografts

All experimental procedures on mice were conducted using a protocol approved by the Augusta University Institutional Animal Care and Use Committee (IACUC). We subcutaneously injected 1 × 10^6^ HT1376 NT (wild type ARRB1) or HT1376 ARRB1 KO (clone 10) into eight week old athymic nu/nu female mice (Envigo Inc, Harlan, IN, USA). We monitored tumor growth weekly. Tumor volume was measured three times per week with a handheld manual caliper (Tumorimeter and RECIST Caliper [Cancer Technologies Inc., Toronto, ON, Canada]) and tumor volume was calculated using the formula: (width)^2^ × length/2 [25]. Animal weight was monitored weekly.

### 2.11. Statistical Analysis

All in vitro data were performed three times independently, unless indicated otherwise. One representative experiment for each assay is shown. We analyzed the significance of data using the built-in statistical program in Graph Pad Prism analysis tool (Graph Pad Inc., version 9, San Diego, CA, USA).

## 3. Results

### 3.1. ARRB1 Depleted Clones Show Decrease Spheroid Formation Potential and Tumorigenicity

**Figure 1:**
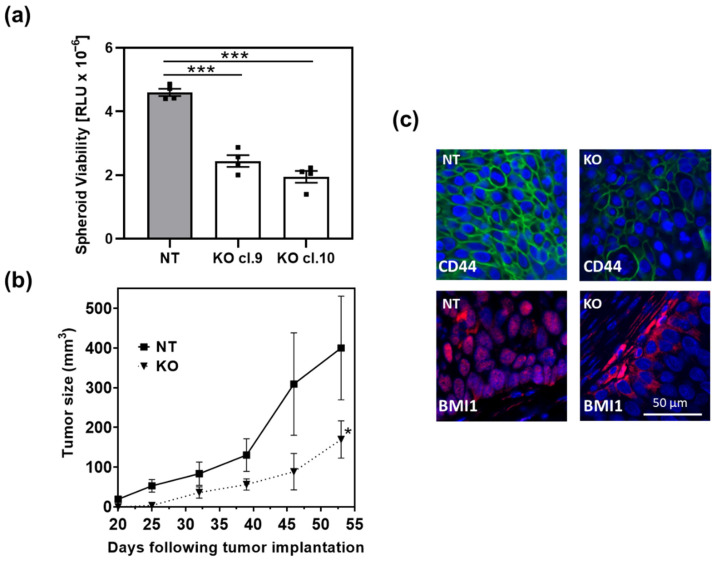
ARRB1 depletion affects spheroid formation and tumor growth: ARRB1 KO clones were established using the CRISPR/Cas9 technology as described previously [19]. (**a**) ARRB1-depleted HT1376 cells (KO cl.9 and KO cl. 10) and corresponding ARRB1 wild type controls (NT) were seeded at 2000 cells/well in 96-well plates for spheroid formation. Cells were placed on SeedEZ scaffolds to prevent attachment on plates and to allow spheroid formation as described in Material and Methods. The Cell Titer-Glo 3D Cell Viability assay was utilized to determine spheroid viability. Data: means ± SEM, *n* = 4; *** *p* < 0.001 (one-way ANOVA). (**b**) 1 × 10^6^ ARRB1-depleted HT1376 cells (KO cl.10) (*n* = 6) and corresponding controls (NT) (*n* = 6) were transplanted subcutaneously in nude mice. Tumor growth was monitored weekly. Data: means ± SEM; * *p* = 0.0459 (two-way ANOVA). (**c**) Detection of CSC markers Bmi-1 and CD44 in ARRB1-KO HT1376 tumor xenografts and corresponding controls (NT) using immunofluorescence. Scale bar: 50 µm.

We previously generated HT1376 ARRB1 knock out (KO) clones utilizing the CRISPR/Cas9 technology [19]. Depletion of ARRB1 abrogated stem cell markers CD44 and Bmi-1 [19]. In the present work we first analyzed the effect of ARRB1 depletion on sphere formation potential in vitro (Figure 1a). ARRB1 KO clones showed decreased spheroid formation potential (Figure 1a), consistent with our previous observations of reduced CSC markers in ARRB1 KO cells. In accordance with this observation, ARRB1 KO clones exhibited significantly slower tumor growth in nude mice (Figure 1b). Consistently with our previous in vitro data, CD44 was depleted in ARRB1 KO tumors, and Bmi-1 showed a cytoplasmic localization in contrast to the corresponding controls (NT) where Bmi-1 was found in the nucleus (Figure 1c). In summary, our data show that ARRB1 is an important regulator of CSC characteristics and tumor cell growth in attachment independent conditions and in vivo. We next sought whether the differential growth in the absence of ARRB1 is attributed to metabolic reprogramming in ARRB1-depleted cells.

### 3.2. Depletion of ARRB1 in CSC-Like Cells Results in Metabolic Reprogramming

**Figure 2:**
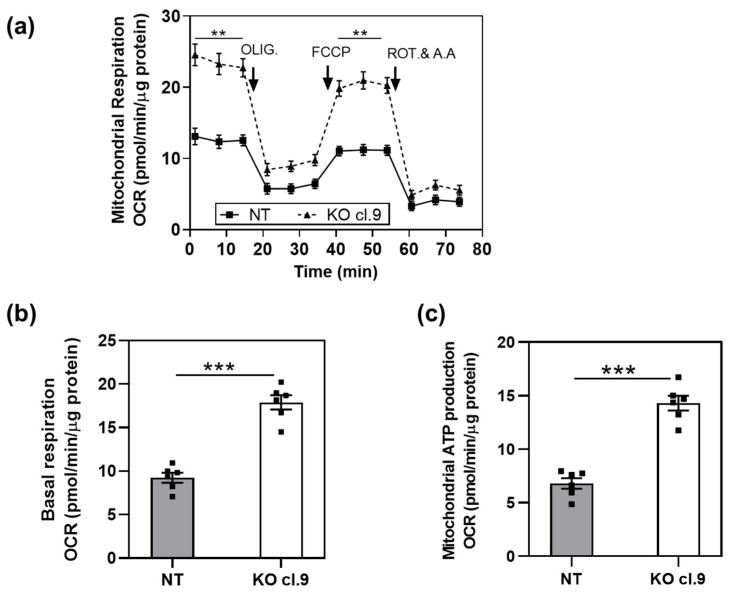
Depletion of ARRB1 alters mitochondrial respiration: (**a**) oxygen consumption rate (OCR) of ARRB1-depleted HT1376 cells (KO cl.9) and corresponding controls (NT) prior to and after injection of respiration modulators oligomycin (OLIG.), FCCP, and rotenone/antimycin A (ROT and A.A). Data: means ± SEM, *n* = 6; ** *p* < 0.01 (Two-way RM ANOVA). (**b**) Basal respiration in ARRB1-depleted HT1376 cells (KO cl. 9) and corresponding controls (NT). Data: means ± SEM, *n* = 6; *** *p* < 0.001 (two-tailed unpaired *t* test). (**c**) Mitochondrial ATP production in ARRB1-depleted HT1376 cells (KO cl. 9) and corresponding controls (NT) (calculated using the Seahorse XF Cell Mito Stress Test Generator). Data: means ± SEM, *n* = 6; *** *p* < 0.001 (two-tailed unpaired *t* test).

In our previous work, we showed that the BC cell line HT1376 expresses CSC markers and forms spheres when serially passaged in stem cell media at low adhesion conditions, indicating a CSC-like phenotype [19]. Moreover, we showed that depletion of ARRB1 (ARRB1 KO clones) resulted in abrogation of CSC markers such as CD44 and Bmi-1 [19]. Since stem cells and CSCs are characterized by different metabolic programs compared to the nonstem cell populations, we investigated the metabolic preference of ARRB1 KO cells compared to corresponding controls. First, we performed mitochondrial stress experiments and tested oxygen consumption (OCR) in ARRB1 depleted HT1376 and control cells. The OCR was measured in ARRB1-depleted cells under both basal and FCCP-stimulated conditions (Figure 2a). Basal OCR and mitochondrial ATP production during basal respiration were increased in ARRB1 KO cells compared to corresponding controls (Figure 2b,c).

**Figure 3:**
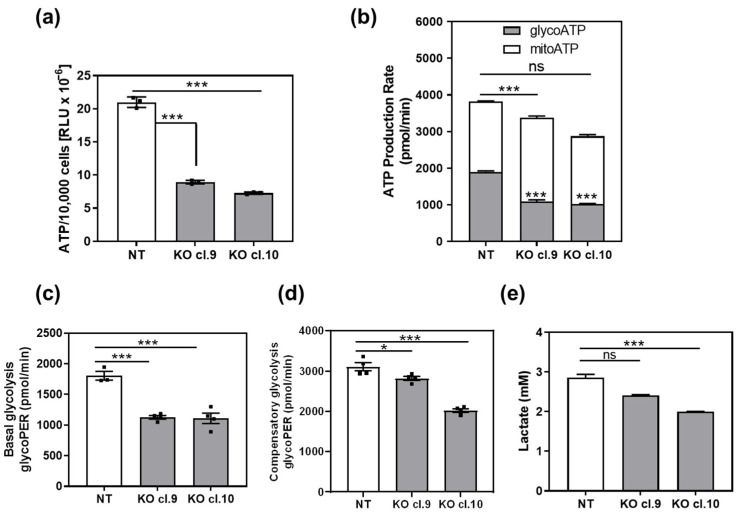
Depletion of ARRB1 decreases glycolysis and glycolytic ATP production rate: (**a**) total ATP per 10,000 cells in ARRB1-depleted HT176 clones (KO cl. 9 and cl.10) compared to control cells (NT). Data: means ± SEM, *n* = 3; *** *p* < 0.001 (One-way ANOVA). (**b**) Calculated glycolytic and mitochondrial ATP production rate in ARRB1-depleted HT1376 cells (KO cl. 9 and cl.10) compared to corresponding controls (NT). Data: means ± SEM, *n* = 3 or 4; *** *p* < 0.001 (glycoATP, KO vs. NT), *** *p* < 0.001 (mitoATP, KO vs. NT) (Two-way ANOVA). Total ATP: (NT vs. Cl.9, ** *p* < 0.01; NT vs. Cl.10, *** *p* < 0.001) (**c**) Basal glycolytic proton efflux rate (PER) in ARRB1 depleted HT1376 cells (KO cl. 9 and cl.10) compared to corresponding controls (NT). Data: means ± SEM, *n* = 3 or 4; *** *p* < 0.001, (One-way ANOVA). (**d**) Glycolytic proton efflux rate (glycoPER) following treatment with inhibitors of oxidative phosphorylation (Rotenone and Antimycin A). Data: means ± SEM, *n* = 4; * *p* < 0.05; *** *p* < 0.001 (one-way ANOVA). (**e**) Quantification of lactate secreted by ARRB1-depleted cells (KO cl. 9 and cl.10) and corresponding controls (NT). Data: means ± SEM, *n* = 7 or 8; *** *p* < 0.001 (One-way ANOVA).

Mitochondrial oxidative phosphorylation and glycolysis are the two major metabolic pathways responsible for ATP synthesis. We first measured total ATP levels in ARRB1 KO clones and corresponding controls (Figure 3a). The total ATP level was significantly lower in ARRB1 KO cells compared to controls (Figure 3a). In parallel experiments, we measured glycolytic and mitochondrial ATP production rate (Figure 3b). The mitochondrial ATP rate was slightly increased in ARRB1-KO clones. In contrast, the glycolytic ATP rate was significantly decreased in ARRB1-KO clones compared to controls (Figure 3b). Moreover, these results confirm a decrease in the total ATP synthesis rate upon abrogation of ARRB1 (Figure 3b).

To determine the glycolytic rate in ARRB1-KO clones and corresponding controls, we calculated the glycolytic proton efflux rate in parallel experiments (Figure 3c). These data were calculated by subtracting the contribution of mitochondrial CO_2_ to extracellular acidification from the total proton efflux rate. ARRB1-KO clones (cl.9 and cl.10) exhibit significantly decreased basal glycolysis. Furthermore, we measured the glycolytic rate following treatment with mitochondrial inhibitors inhibiting oxidative phosphorylation (Rotenone and Antimycin A) to calculate compensatory glycolysis (Figure 3d). The latter indicates the potential of cells to switch to glycolysis to meet energy demands when mitochondrial respiration is blocked. We observed reduced compensatory glycolysis in ARRB1-KO clones compared to corresponding controls. Further, we measured produced and secreted L-lactate as a measure for glycolysis (Figure 3e). We observed significantly decreased extracellular lactate concentration in ARRB1 depleted cells (cl. 9 and cl. 10) compared to corresponding controls (NT), confirming our observations from the glycolytic rate assays. Taken together, our results demonstrate that ARRB1 depletion in BC cells resulted in increased oxidative phosphorylation and decreased glycolysis along with cellular ATP levels.

### 3.3. ARRB1 Regulates Pyruvate Transport to the Mitochondria and Glucose Uptake in Cells

**Figure 4:**
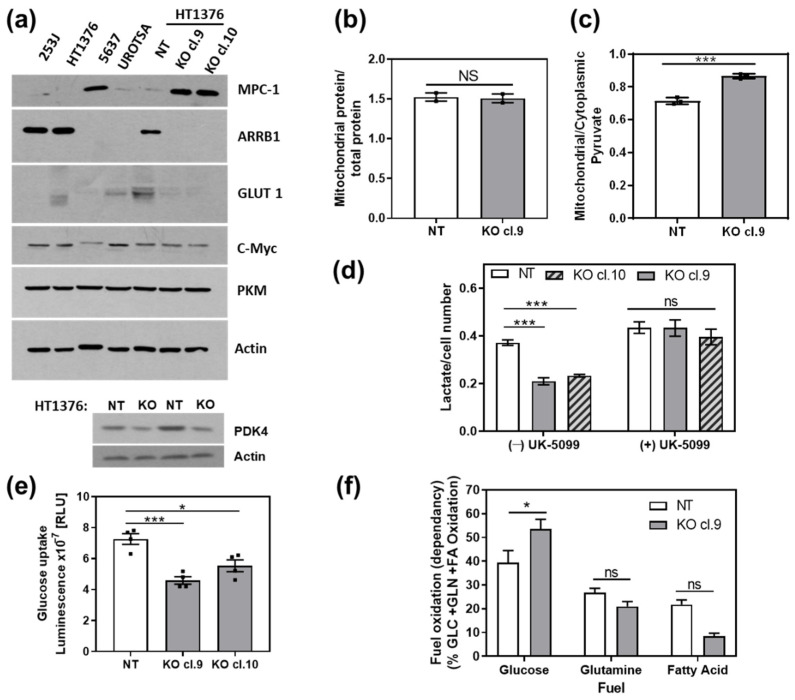
ARRB1 regulates mitochondrial translocation of pyruvate and glucose uptake: (**a**) immunoblot for detection of glycolytic markers, MPC1 and GLUT1 in bladder cancer cell lines (253J, HT1376, 5637) and nonmalignant cells (UROTSA), as well as ARRB1-depleted HT1376 cells (KO cl. 9 and cl. 10). (**b**) Mitochondria of ARRB1-depleted HT1376 cells (KO cl. 9) and corresponding controls (NT) were collected, and mitochondria/total protein ratio was calculated. Data: means ± SEM, *n* = 2. The experiment was repeated once (**c**) Mitochondrial and cytoplasmic extracts were collected for colorimetric detection of pyruvate. Data: means ± SEM, *n* = 3; *** *p* < 0.001 (two-tailed unpaired *t* test). The experiment was repeated once (**d**) Secreted Lactate (glycolysis) in presence or absence of the MPC1-inhibitor UK-5099. Data: means ± SEM, *n* = 4; *** *p* < 0.001 (two-way ANOVA). (**e**) Glucose uptake in ARRB1-depleted HT1376 cells (KO cl. 9 and cl.10) compared to corresponding controls (NT). Data: means ± SEM, *n* = 4; *** *p* < 0.001, * *p* < 0.05 (One-way ANOVA). (**f**) Measurement of mitochondrial fuel usage dependency of ARRB1-depleted HT1376 cells to oxidize glucose/pyruvate, glutamine/glutamate, and long-chain fatty acids. Data: means ± SEM, *n* = 4 to 8; * *p* < 0.05, (two-way ANOVA). NS: not significant.

Our results suggest that ARRB1 regulates the metabolic preference of BC CSC-like cells since depletion of ARRB1 resulted in increased oxidative phosphorylation and decreased glycolysis. We further aimed to elucidate how ARRB1 regulates metabolic pathways. First, we looked at the expression of ARRB1, glycolytic enzymes and the mitochondrial pyruvate carrier MPC1 in several BC cell lines. Interestingly, ARRB1 was differentially expressed in BC cell lines and its expression inversely correlated with the expression of MPC1 (253J, HT1376, 5637) (Figure 4a). Importantly, the depletion of ARRB1 in HT1376 cells (KO cl. 9, cl. 10) resulted in a dramatic increase in MPC1 compared to corresponding controls (NT, wildtype ARRB1) (Figure 4a and Figure S2).

The depletion of ARRB1 (cl. 9, cl. 10) did not affect the expression of the glycolytic enzyme PKM and c-MYC, a transcription factor involved in glycolysis (Figure 4a). Furthermore, depletion of ARRB1 in HT1376 cells (KO cl.9 and 10) resulted in an abrogation of the glucose transporter GLUT 1 (Figure 4a). Moreover, ARRB1 KO cells showed reduced levels of pyruvate dehydrogenase kinase 4 (PDK4) (Figure 4a, lower panel). In conclusion, our results show that ARRB1 depletion decreases GLUT1 and PDK4 and induces MPC1 protein levels.

MPC1 is a key metabolic regulator at the crossroad between oxidative phosphorylation and glycolysis [26]. MPC1 translocates pyruvate into the mitochondria which becomes converted to Acetyl-CoA that feeds to the TCA followed. First, we analyzed mitochondrial biogenesis as a cause for the increased MPC1 expression in ARRB1 depleted cells. The mitochondrial/total protein ratio did not change in ARRB1 depleted cells compared to controls, suggesting no change in mitochondrial number (Figure 4b). However, the depletion of ARRB1 resulted in an increase in mitochondrial pyruvate levels (Figure 4c), consistent with the increased expression of MPC1 (Figure 4a) and increased mitochondrial respiration (Figure 2a) in ARRB1 KO cells. As shown in Figure 3, ARRB1 KO cells showed reduced glycolysis compared to controls. We further aimed to decipher the role of ARRB1 in regulating glycolysis. We treated ARRB1 KO cells and corresponding controls with the MPC1 inhibitor UK-5099 and analyzed glycolysis by measuring extracellular lactate levels (Figure 4d). In the absence of UK-5099 ((−) UK-5099), we observed reduced lactate levels/glycolysis in ARRB1 KO cells compared to controls (NT). However, treatment with UK-5099 restored lactate production/glycolysis in ARRB1 KO clones (Figure 4d). These results indicate that MPC1 mediates the effects of ARRB1 on glycolysis. Furthermore, we looked at the glucose uptake in ARRB1-KO cells (Figure 4e). The depletion of ARRB1 resulted in decreased glucose uptake compared to controls (Figure 4e), consistent with the reduced levels of GLUT1 in ARRB1 KO cells (Figure 4a). Taken together, the depletion of ARRB1 resulted in an increased expression of MPC1 and mitochondrial transport of pyruvate. In contrast, the protein levels of GLUT 1 and glucose uptake were reduced upon depletion of ARRB1. In parallel experiments, we over expressed ARRB1 in other BC cell lines (5637 and T24) which are characterized by low/not detectable expression of ARRB1. Consistently, over expression of ARRB1 increased GLUT1 protein levels along with glycolysis (lactate production) in both 5637 and T24 cell lines (Figures S1 and S4).

### 3.4. Effect of ARRB1 on the Utilization of Glucose, Glutamine, and Fatty Acids as Mitochondrial Fuels

We showed that depletion of ARRB1 reduced glycolytic rate and prompted cells to reprogram their metabolic pathways towards increased mitochondrial oxygen consumption. Next, we sought to decipher the contribution of glucose/pyruvate, glutamine/glutamate, and fatty acids to the mitochondrial TCA cycle for energy generation of ARRB1-KO vs. control cells (Figure 4f). ARRB1-KO cells exhibit increased glucose-derived pyruvate utilization to fuel the TCA cycle (Figure 4f) consistent with the increased uptake of pyruvate compared to corresponding controls (Figure 4c). Control cells (NT) cells show higher oxidation of glutamine/glutamate and fatty acids compared to ARRB1-KO cells. In conclusion, the depletion of ARRB1 results in a decrease in fatty acid and glutamine oxidation, whereas the dependency of cells on glucose/pyruvate for maintaining baseline respiration increases.

### 3.5. Over Expression of ARRB1 in ARRB1 KO Cell Lines Restores the Metabolic Program

**Figure 5:**
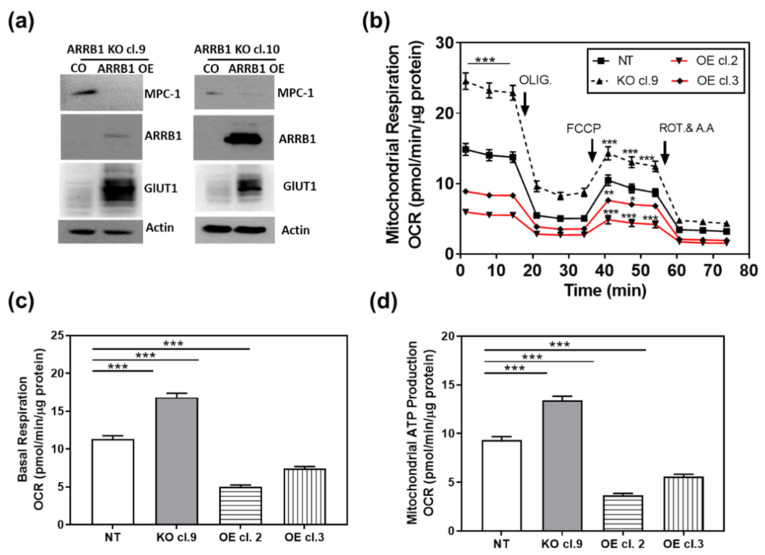
Re-expression of ARRB1 in ARRB1 depleted cells restores the metabolic program: (**a**) HT1376 ARRB1 KO cells (KO cl. 9 and KO cl. 10) were transfected with ARRB1 for re-expression/overexpression (OE) of ARRB1. Protein extracts were isolated for detection of ARRB1 and MPC1 by Western blot analysis. (**b**) ARRB1 KO cells (KO cl. 9) were transfected with ARRB1, and stable clones overexpressing ARRB1 were generated (OE cl.2 and cl.3). The Metabolic preference of clones was analyzed by measuring the oxygen consumption rate. Data: means ± SEM, *n* = 8; *** *p* < 0.001, ** *p* < 0.01,* *p* < 0.05 (two-way ANOVA). (**c**,**d**) Basal respiration and mitochondrial ATP production in clones generated following re-expression/overexpression of ARRB1. Data: means ± SEM, *n* > 10; *** *p* < 0.001 (one-way ANOVA).

We performed proof-of-principle experiments and overexpressed ARRB1 in ARRB1-KO clones (Figure 5a). When ARRB1 was overexpressed, the phenotype was reversed and cells exhibited decreased expression of MPC1 (Figure 5a and Figure S3). In parallel experiments, we over expressed ARRB1 in ARRB1 KO cells (cl. 9) and generated stable clones overexpressing ARRB1 (OE cl.2, cl. 3). We then analyzed mitochondrial respiration in those clones (Figure 5b). ARRB1 KO clones (cl. 9) showed increased mitochondrial respiration compared to NT controls (Figure 2a and Figure 5b). The overexpression of ARRB1 in ARRB1 KO cells (OE cl.2, cl.3) reduced mitochondrial respiration/oxygen consumption. Similarly, Figure 5c,d show reduced basal respiration and mitochondrial ATP production, respectively, when ARRB1 is re-expressed in ARRB1 KO cells. In conclusion, ARRB1 depletion results in metabolic reprogramming towards mitochondrial respiration/oxidative phosphorylation. However, the metabolic preference is restored when ARRB1 is re-expressed.

## 4. Discussion

In the current work, we show for the first time that a scaffold protein, ARRB1, functions as a metabolic switch regulating metabolic reprograming in CSC-like BC cells. Depletion of ARRB1 resulted in a dramatic increase in MPC1—a pyruvate carrier that couples glycolysis and oxidative phosphorylation—and elevated mitochondrial pyruvate levels, which are associated with increased oxygen consumption/mitochondrial respiration. Importantly, ARRB1 depletion abrogated glucose transporter GLUT1 levels and significantly inhibited glucose uptake and glycolysis. These results strongly suggest that ARRB1 dictates the metabolic phenotype in CSC-like BC by regulating MPC1 and GLUT1 and thus glycolysis. GLUT1 plays a key role in bladder cancer development [27], and while it is not expressed in normal urothelium, it is over expressed in noninvasive and muscle-invasive bladder cancers. Importantly, GLUT1 expression correlates with the progression of muscle-invasive carcinoma and is associated with poor prognosis [27,28,29,30,31]. Thus, ARRB1-mediated regulation of GLUT1 highlights the role of ARRB1 as a tumor oncogene in BC.

Consistent with our data, Zecchini et al. showed that ARRB1 potentially promotes aerobic glycolysis when accumulated in the nucleus of prostate cancer cells. The authors demonstrated that nuclear ARRB1 promoted HIF1α transcriptional activity under normoxic conditions, a phenomenon called pseudohypoxia [32]. Specifically, nuclear ARRB1 resulted in an increase in fumarate and succinate concentrations that indirectly promoted transcriptional activity of HIF1α by inhibition of the PHD proteins (negative regulators of HIF1α). In accordance with our results, the authors showed that nuclear ARRB1 promoted glucose uptake and aerobic glycolysis, which were reduced when ARRB1 was knocked down [32]. Interestingly, the authors observed an increase in TCA cycle metabolite level in prostate cancer cells with nuclear localization of ARRB1. However, the authors demonstrated that glucose was not the major fuel/carbon source for the TCA. In the current work, we showed that in BC cells expressing high levels of ARRB1, glucose was the major fuel of the TCA compared to glutamine and fatty acids. However, when ARRB1 was depleted, the usage of glucose as a TCA fuel was further increased. These results suggest that ARRB1 negatively regulates the utilization of glucose in aerobic respiration. Furthermore, our data show that ARRB1 inhibits MPC1 and mitochondrial translocation of pyruvate and therefore utilization of the glucose-derived metabolite pyruvate as a fuel for the TCA.

We have previously shown that depletion of ARRB1 abrogates the expression of stem cell markers (e.g., CD44, Bmi-1) and spheroid formation potential, suggesting the importance of ARRB1 in regulating a SC phenotype [19]. A potential role of ARRB1 in maintaining SC characteristics has also been demonstrated in nonsmall cell lung cancer cells [33]. Perumal et al. showed that nicotine drives the expression of stem cell factor (SCF) in an ARRB1-dependent manner and promotes self-renewal in the side-population stem cell-like cells in nonsmall cell lung cancer [33]. Stem cells are often characterized by differential metabolic programs and our work shows for the first time that ARRB1 regulates the metabolic preferences of CSC-like BC cells by promoting glycolysis.

Recent studies have suggested that MPC1 functions as a repressor of the Warburg effect in colon cancer and is a marker that inversely correlates with stem cell properties [17]. MPC1 is downregulated or depleted in a plethora of cancers [17,34]. Low MPC1 levels correlate with a poor prognosis in several cancers, including renal cell carcinoma [17,35]. Consistently, recent evidence has shown that MPC1-mediated regulation of mitochondrial pyruvate metabolism controls stem cell properties and proliferation in intestinal cells [36].

In the current work, we show for the first time that the scaffold protein ARRB1 negatively regulates MPC1. Our ARRB1 KO clones were characterized by increased MPC1 levels, which favor mitochondrial uptake of pyruvate and oxidative phosphorylation in more differentiated cells. In the contrary, lower MPC1 levels promote a glycolytic program associated with more rapid tumor growth in our model, consistent with observations from colon cancer cells [17,34]. The current work shows that ARRB1 functions as a molecular switch that promotes stem cell properties by mediating the metabolic reprogramming towards glycolysis favored by many proliferative cancer cells and (cancer) stem cells.

## 5. Conclusions

In conclusion, the current work demonstrates for the first time that the scaffold protein ARRB1 promotes a metabolic program that relies on glycolysis for ATP generation. Our work opens new avenues for the treatment of CSC-like cells in BC. However, our data show that abrogation of ARRB1 in BC cells shifts the metabolic program towards mitochondrial oxidative phosphorylation, underlining the metabolic flexibility of cancer (stem cell-like) cells. Thus, future therapies targeting ARRB1 would benefit from a combination of treatments targeting ARRB1 with inhibitors of mitochondrial cancer cell metabolism to combat the metabolic adaptation potential of cancer cells.

## Data Availability

Data is contained within the article or Supplementary Materials.

## Supplementary Materials

The following are available online at https://www.mdpi.com/article/10.3390/cancers13081809/s1, Figure S1: Over expression of ARRB1 in bladder cancer cell lines 5637 and T24 increases glycolysis, Figure S2: uncropped western blots from Figure 4, Figure S3: uncropped western blots from Figure 5, Figure S4: uncropped western blots from Supplementary Figure S1, Table S1: Antibodies and dilutions.
