# Supplementary material for: ARRB1 Regulates Metabolic Reprogramming to Promote Glycolysis in Stem Cell-Like Bladder Cancer Cells
Source: Cancers (Basel). 2021 Apr 10;13(8):1809. doi: 10.3390/cancers13081809 (PMC8069028; doi:10.3390/cancers13081809)
Supplement: Supplementary file 1 [file cancers-13-01809-s001.pdf]

cancers MDPI

Supplementary Material: ARRB1 Regulates Metabolic Reprogramming to Promote Glycolysis in Stem Cell-Like Bladder Cancer Cells

Kenza Mamouni, Jeongheun Kim, Bal L. Lokeshwar and Georgios Kallifatidis

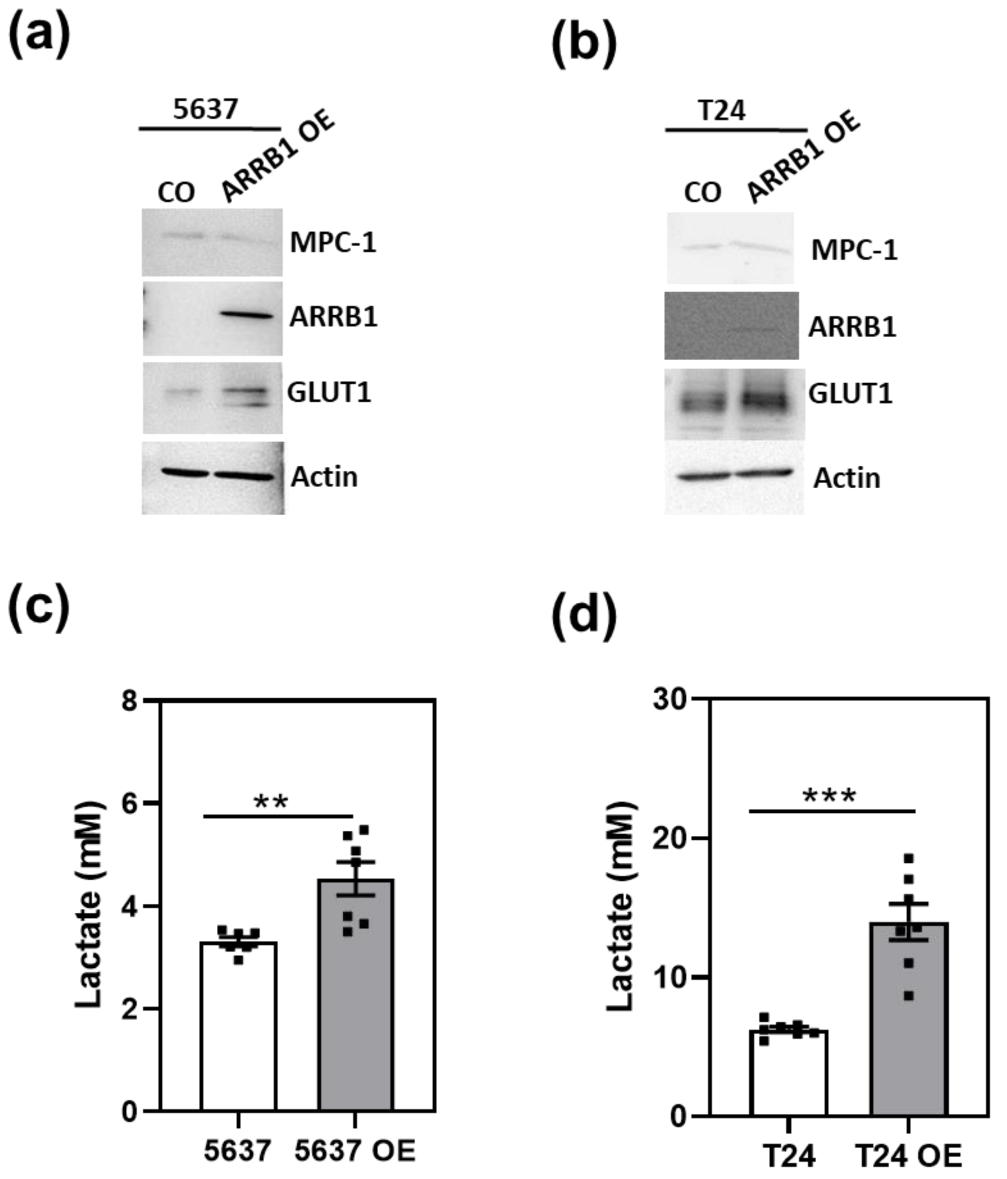

Figure S1. Over expression of ARRB1 in bladder cancer cell lines 5637 and T24 increases glycolysis: **(a, b)** Immunoblot for detection of MPC1 and GLUT1 in bladder cancer cell lines (5637 and T24) following over expression of ARRB1. **(c, d)** Quantification of glycolysis (lactate production) in ARRB1-overexpressing cells (5637 OE and T24 OE) and corresponding controls. Data: means ± SEM, n=6 or 7; *** $p<0.001$, ** $p<0.01$ (two-tailed unpaired t test). The experiments were repeated once.

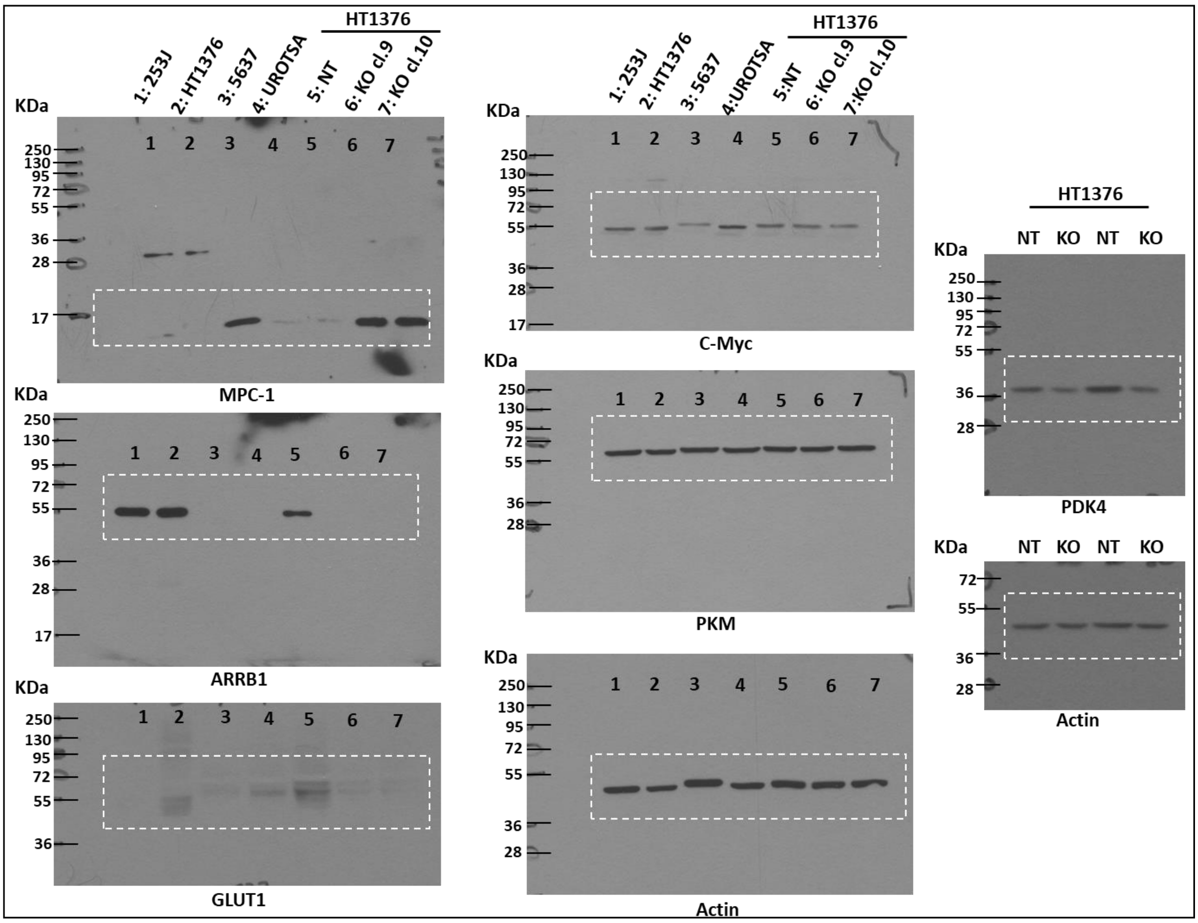

Figure S2. uncropped western blots from Figure 4.

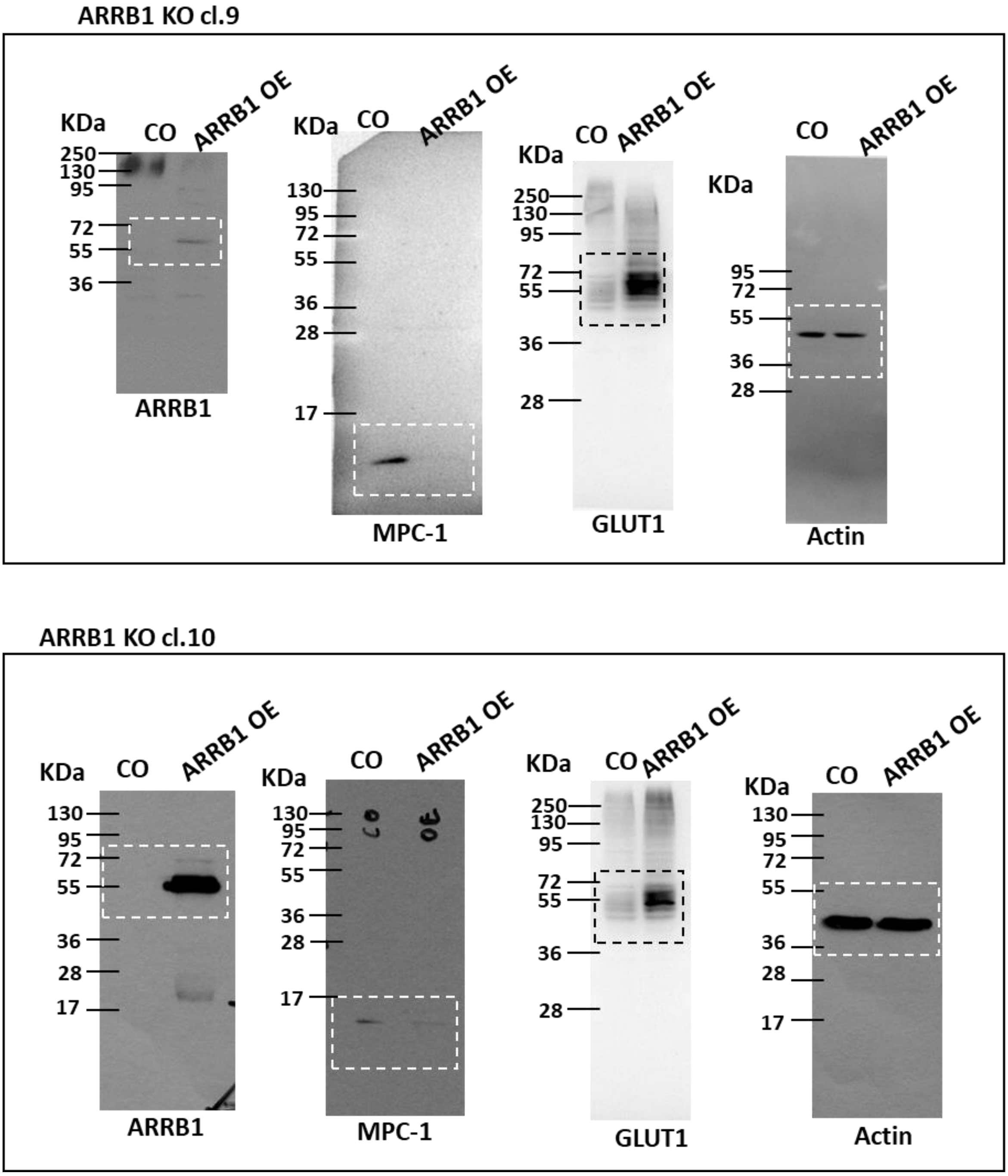

Figure S3: uncropped western blots from Figure 5

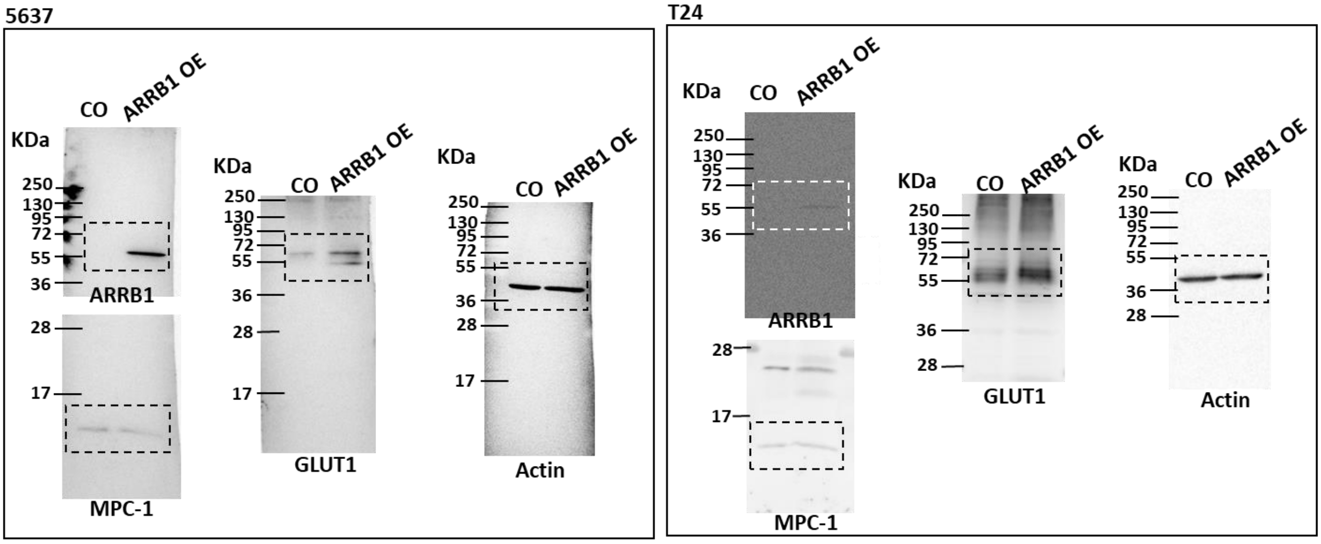

Figure S4: uncropped western blots from Supplementary Figure S1.

Table S1: Antibodies and dilutions

Antibodies used in Western Blot analysis					
**Target**	**Host Species**	**Supplier**	**Catalog number**	**Dilution**	**Blocker**
**Actin (13E5)**	rabbit	Cell Signaling	5125s	1:100,000	5% NFDM
**ARRB1 (D803J)**	rabbit	Cell Signaling	12697s	1:1,000	5% NFDM
**c-Myc (Lot# 1151610301)**	rabbit	ABclonal	A1309	1:3,000	5% NFDM
**Glut 1 (D3J3A)**	rabbit	Cell Signaling	12939s	1,000-1:3000	5%BSA
**MPC1 (D2L9I)**	rabbit	Cell Signaling	14462s	1:1,000	5% Milk
**PDK4 (Lo# 3301330101)**	rabbit	ABclonal	A3069	1:5,000	5% Milk
**PKM**	rabbit	ABclonal	A0268	1:3,000	5% Milk

Antibodies used in Immunofluorescence					
**Target**	**Host Species**	**Supplier**	**Catalog number**	**Dilution**	**Blocker**
**Bmi1 (D20B7) XP (R)**	rabbit	Cell Signaling	6964s	1:200	3% BSA, 10% goat serum
**HCAM/CD44 (DF 1485)**	mouse	Santa Cruz Bio-technology	Sc-7297	1:50	3% BSA, 10% goat serum
